# Genomic diversity in pearl millet inbred lines derived from landraces and improved varieties

**DOI:** 10.1186/s12864-020-06796-4

**Published:** 2020-07-08

**Authors:** Ghislain Kanfany, Desalegn D. Serba, Davina Rhodes, Paul St. Amand, Amy Bernardo, Prakash I Gangashetty, Ndjido Ardo Kane, Guihua Bai

**Affiliations:** 1 Institut Sénégalais de Recherches Agricoles (ISRA), Centre National de Recherches Agronomiques de Bambey, Diourbel, Senegal; 2 Agricultural Research Center-Hays, Kansas State University, 1232 240th Avenue, Hays, KS 67601 USA; 3 Department of Agronomy, Kansas State University, Manhattan, KS USA; 4 Hard Winter Wheat Genetics Research Unit, USDA-ARS, Manhattan, KS USA; 5 International Crops Research Institute for the Semi-Arid Tropics (ICRISAT), Niamey, Niger; 6 Institut Sénégalais de Recherches Agricoles/Centre d’Étude Régional pour l’Amélioration de l’Adaptation à la Sécheresse (ISRA/CERAAS), Thiès, Senegal

**Keywords:** Genetic diversity, Population structure, *Pennisetum glaucum*, *Cenchrus americanus*, Genotyping-by-sequencing, SNPs, Linkage disequilibrium

## Abstract

**Background:**

Genetic improvement of pearl millet is lagging behind most of the major crops. Development of genomic resources is expected to expedite breeding for improved agronomic traits, stress tolerance, yield, and nutritional quality. Genotyping a breeding population with high throughput markers enables exploration of genetic diversity, population structure, and linkage disequilibrium (LD) which are important preludes for marker-trait association studies and application of genomic-assisted breeding.

**Results:**

Genotyping-by-sequencing (GBS) libraries of 309 inbred lines derived from landraces and improved varieties from Africa and India generated 54,770 high quality single nucleotide polymorphism (SNP) markers. On average one SNP per 29 Kb was mapped in the reference genome, with the telomeric regions more densely mapped than the pericentromeric regions of the chromosomes. Population structure analysis using 30,208 SNPs evenly distributed in the genome divided 309 accessions into five subpopulations with different levels of admixture. Pairwise genetic distance (GD) between accessions varied from 0.09 to 0.33 with the average distance of 0.28. Rapid LD decay implied low tendency of markers inherited together. Genetic differentiation estimates were the highest between subgroups 4 and 5, and the lowest between subgroups 1 and 2.

**Conclusions:**

Population genomic analysis of pearl millet inbred lines derived from diverse geographic and agroecological features identified five subgroups mostly following pedigree differences with different levels of admixture. It also revealed the prevalence of high genetic diversity in pearl millet, which is very useful in defining heterotic groups for hybrid breeding, trait mapping, and holds promise for improving pearl millet for yield and nutritional quality. The short LD decay observed suggests an absence of persistent haplotype blocks in pearl millet. The diverse genetic background of these lines and their low LD make this set of germplasm useful for traits mapping.

## Background

Pearl millet (*Pennisetum glaucum* (L.) R. Br. syn. *Cenchrus americanus* (L.) Morrone) is a climate-resilient crop grown in the arid and semi-arid areas of the world. Pearl millet is the most widely grown millet species, accounting for approximately half of the total worldwide production of millets [36]. It has been traditionally grown for thousands of years for human consumption in dry areas of Africa and Asia for its higher nutritive values compared to other cereals [2]. It is well adapted to the Sahelian and Sudanian agro-ecosystems in West and Central Africa (WCA) where other cereals have difficulty growing in the harsh environment. It has a very efficient energy production system to adapt to the hot and dry climates [39], and is even more tolerant to drought than sorghum [12].

Distressed crop production under suboptimal environmental conditions owing to global climate change is an inevitable scenario. Therefore, on top of the currently estimated 41% of the land area of arid and semiarid zones considered unsuitable for crop production [30], more land is expected to become unfavorable for crop production. Research emphasis on climate-smart crops such as pearl millet would have significant contribution in mitigation of the negative impacts of the looming climate change on food security of the dissolute environments.  Although pearl millet is grown as a rainfed crop in a wide range of ecological zones and production systems, its yield is very low and spatially and temporally variable. This low productivity is predominantly attributed to limited genetic improvement and availability of improved varieties [38], along with agronomic and socio-economic production constraints. An innovative breeding strategy is required to develop improved climate-resilient pearl millet cultivars that can contribute to a sustainable food supply for the ever-increasing population [33].

Pearl millet is also gaining a reputation as a health-promoting nutritious grain. The grain contains vital nutrients and is considered to be equal or superior to that of wheat (*Triticum aestium* L.), maize (*Zea mays* L.), sorghum (*Sorghum bicolor* Moench), and rice (*Oryza sativa* L.) in its nutritional value [20]. It is an important source of dietary energy, and provides nutritional security for people in the most dissolute regions, particularly in WCA and Indian subcontinent. Previous studies have shown that pearl millet is an excellent source of micronutrients like iron and zinc [22]. Significant genetic variations for mineral densities and moderate heritability [29] warrant a high potential for biofortification and development of nutrient-dense foods. Tapping into this potential of pearl millet for the development of diversified foods with health benefits may provide a low-cost solution for the problems related to micronutrient deficiencies mainly in children and women who are entirely dependent on the crop as a staple food.

Development of molecular markers using next-generation sequencing technology for a breeding population is a very vital tool to expedite pearl millet improvement. High-density molecular markers are critical to quantitative trait loci (QTL) mapping that lays the foundation for marker-assisted breeding to improve the selection efficiency for faster development of cultivars [5, 27]. Molecular markers are also used to investigate genomic-diversity and population structure of crop germplasm for systematic use in new cultivar development and germplasm conservation. With the recent advancements in next-generation sequencing (NGS) and increasingly affordable prices, profiling genome-wide DNA sequence variations, high-resolution QTL mapping, and identification of candidate genes and natural allelic variants for QTLs governing important traits have become routine practices for many crops.

Characterization of the genetic diversity and population structure of pearl millet germplasm and breeding populations is needed in order to accelerate its genetic improvement for agronomic and nutritional traits. Toward this goal, a panel of 309 inbred lines derived from landraces and improved open pollinated varieties collected from different parts of Africa and India were evaluated for population genomics using genotyping-by-sequencing (GBS) markers. The present investigation employed pertinent population genomic approaches to understand the extent of genetic diversity of a pearl millet inbred lines population comprising both potential restorers and seed parents for hybrid variety development.

## Results

### Development of SNP markers

A total of ~ 750 million total unfiltered reads were generated from 309 inbred lines arranged in four GBS plates run twice independently on an Ion Proton Next-Generation Sequencer (ThermoFisher Scientific, Waltham, MA, US). All the raw sequence reads for all the accessions have been submitted to the National Center for Biotechnology Information (NCBI) sequence read archive and deposited under the accession ID “BioProject ID”: PRJNA598172. Mapping the GBS reads to the pearl millet reference genome sequence initially detected 150, 977 unfiltered and non-imputed SNPs for the panel. Among these, 123,995 SNPs were distributed on chromosomes 1 to 7. A total of 26,982 SNPs were mostly from the unanchored genome sequences or from the part of the genome not covered in the reference genome sequence. The raw number of SNPs mapped on each chromosome ranged from 13,432 (chr 7) to 21,665 (chr 1).

**Fig. 1:**
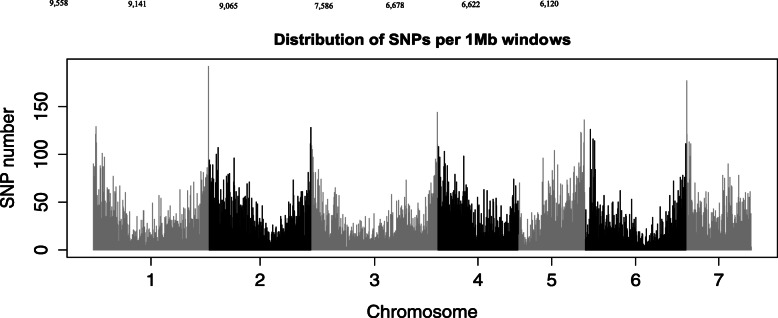
Genome-wide distributions of 54,770 high quality single-nucleotide polymorphism (SNPs) derived from GBS of 309 pearl millet inbred lines. The x-axis represents the lengths of seven chromosomes and the y-axis indicates the number of SNPs in 1 Mb genome windows. The figures above the bars represent the total number of markers mapped on each chromosome

A high-density of high quality SNPs across the seven pearl millet chromosomes with most of the SNPs distributed in the telomeric regions than the pericentromeric regions of the chromosomes (Fig. 1) were visualized. Markers density varied across the genome, ranging from 0 to 192 SNPs per Mb with an average of 29 Kb per SNP. Chromosome-wise markers density varied from 9558 (chr. 1) to 6120 (chr. 7). In chr. 5, fewer markers were mapped in one arm than the other.

**Fig. 2:**
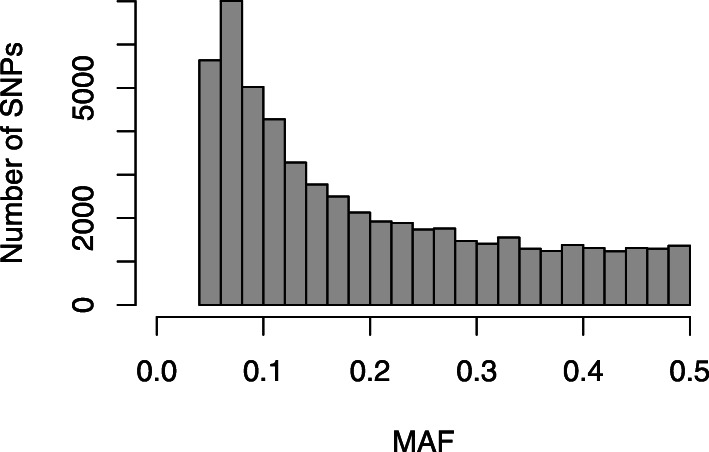
Minor allele frequency (MAF) distribution for 54,770 SNP markers from 309 pearl millet inbred lines using genotyping-by-sequencing (GBS)

Analysis of SNP types showed that transition mutations (33,817, 62%) were much higher than transversion mutations (20,953, 38%) and the transition/transversion ratio was 1.63 (Table 1). In overall, C/T transitions occurred at the highest frequency, while A/T mutation occurred at the lowest frequency among all types of mutations detected. The frequencies were similar between A/G and C/T transitions and among the four transversions, except for A/*T. minor* allele frequency varied from 0.05 to 0.50 with a mean of 0.20 (Fig. 2).

**Table 1:** Transition and transversion mutations of GBS-SNPs detected among 309 pearl millet genotypes

Type of mutation	SNP mutation	Number of SNPs	Total SNPs per type
*Transition*	*A/G*	*16,888*	33,817
	*C/T*	*16,929*	
*Transversion*	*A/T*	*4180*	20,953
	*A/C*	*5434*	
	*C/G*	*5984*	
	*G/T*	*5355*	
Total			54,770

### Genetic diversity

**Fig. 3:**
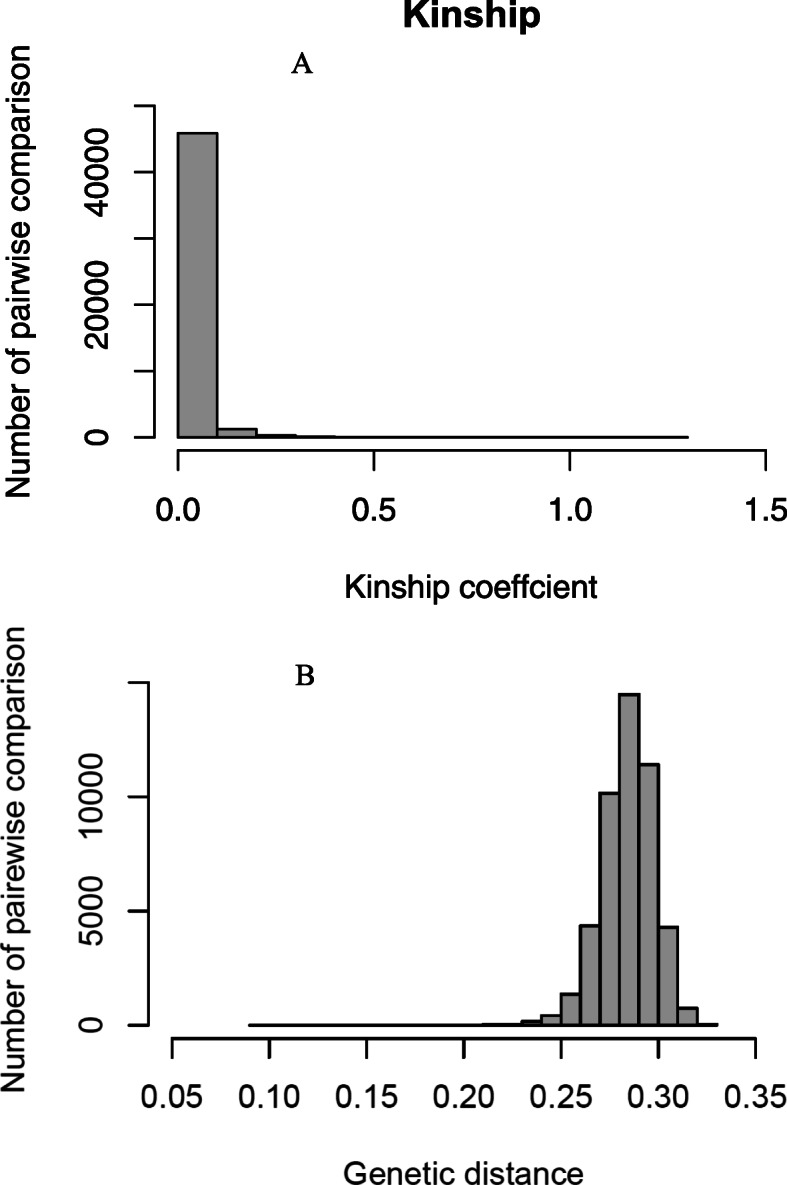
Pairwise relative kinship (**a**) and genetic distance (**b**) of 309 pearl millet genotypes using 54,770 high quality SNP markers developed using genotyping-by-sequencing (GBS)

A total of 54,770 SNPs on the seven chromosomes were used to evaluate genetic diversity in 309 genotypes (Additional file S2). The kinship coefficients between pairs of the 309 inbred lines ranged from 0.00 to 1.21 with a mean value of 0.02 (Fig. 3a). Nearly 91% of the pairwise relative kinship values were close to zero (< 0.05) and the remaining were between 0.05 and 1.21. The highest relative kinship value was observed between ICML197354 (IP-9407) and ICML197438 (IP-17690) derived from landraces collected in Ghana and Togo, respectively (Additional file S3).

The genetic distances (GD) of pairwise comparisons of the inbred lines varied from 0.09 to 0.33 with the average distance of 0.28 (Fig. 3b). The majority of the genetic distances fell between 0.25 and 0.30. The lowest genetic distance (0.09) was observed between ICML197458 (IP-21020) and ICML197279 (IP-4020) developed from landraces collected in Nigeria and India, respectively (Additional file S4). The highest genetic distance (0.33) was observed between inbred ICML197314 (IP-6882) and ICML197390 (IP-11677) derived from landraces.

### Population structure and principal component analysis

**Fig. 4:**
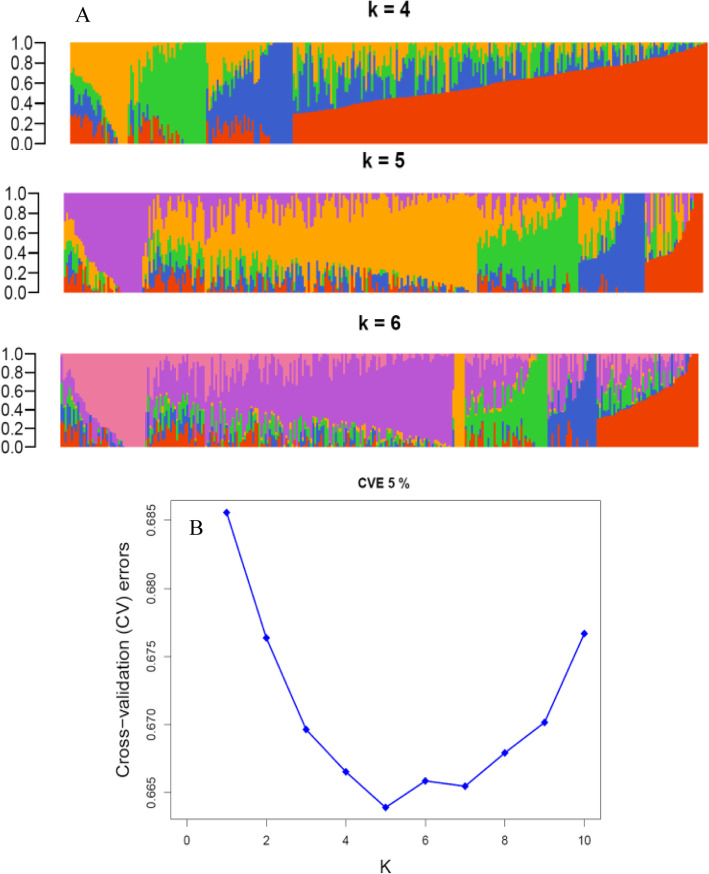
Population structure of 309 pearl millet inbred lines. **a** Bayesian posterior probability of membership determined by the model-based clustering method for hypothetical subpopulations, K values of 1–10. The color of the vertical bar on the x-axis represents the proportion of membership of each inbred line in each subgroup. **b** Cross-validation (CV) errors suggest that the 309 genotypes can be divided into five true genetic populations

Analysis of the pearl millet inbred lines population using 30,893 LD pruned SNPs and the genetic distance matrix of the lines in ADMIXTURE [1] identified five subpopulations (Fig. 4a). The error rate from a cross-validation method used by Admixture to determine the appropriate number of sub-populations rapidly declined from K = 1 to K = 5, indicating that the 309 lines fell into five distinct groups with different levels of admixtures (Fig. 4b). Each line was assigned to a group when the proportion of the membership probability was higher than 0.6. Thus, subgroups 1 and 2 consisted of 33 and 134 lines, whereas subgroups 3, 4 and 5 contained 25, 14 and 10 inbred lines, respectively. The remaining 93 lines were classified as admixtures. No cluster made exclusively of inbred lines from the same country was found. In general, most of the lines closely related in pedigree or derived from landraces/improved varieties with high Fe and Zn content were grouped together, except for subgroup 2 which was formed mainly of inbred lines from the accessions collected in WCA.

**Fig. 5:**
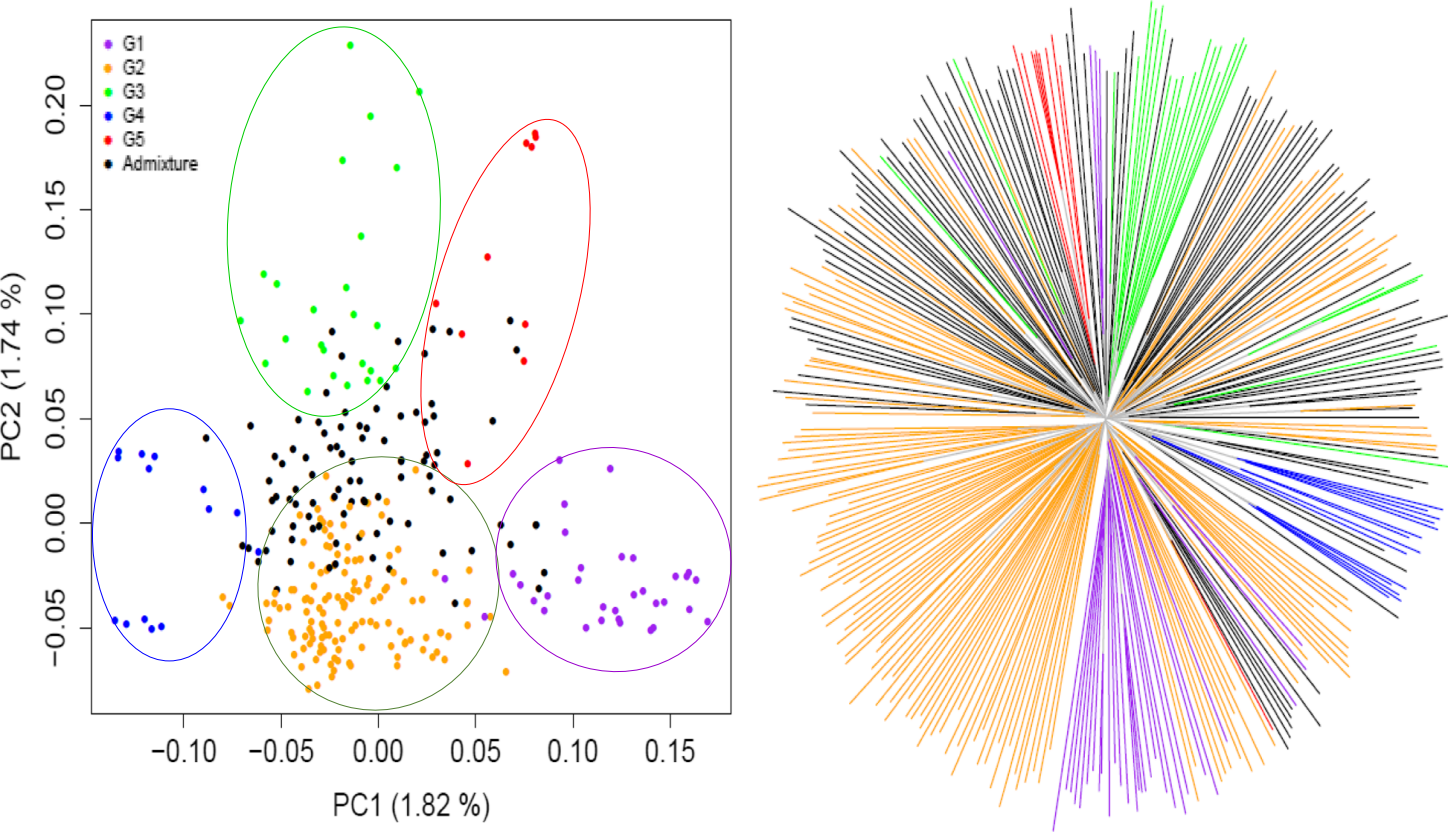
The principal component analysis (**a**) and neighbor joining tree (**b**) of 309 pearl millet inbred lines. The branch colors indicate genotypes corresponding to the subpopulations (Subpopulations 1 to 5) from the population structure analysis in Fig. 4. Five clades were distinguished by distance between branches

To confirm the ADMIXTURE results, principal component analysis (PCA) using the snpgdsPCA function of the R package SNPRelate [41] and genetic relatedness among accessions was visualized using a neighbor joining (NJ) tree which revealed a clear separation between groups. Both the PCA and the NJ tree confirmed the five subgroups found in the population structure analysis (Fig. 5a and b). PCA based on the results of ADMIXTURE revealed that the total genetic variation explained by the first ten PCs was 11.3%. The first and second PCs explained a very low proportion of genetic variation, 1.82 and 1.74%, respectively. The results showed a clear subgroup separation in the panel and agree with the conclusion of five subgroups from ADMIXTURE. Group 3 showed more dispersion than the other groups. Based on genetic distance, the neighbor joining phylogenetic tree also displayed similar subgroups as shown by the ADMIXTURE and PCA analyses.

**Fig. 6:**
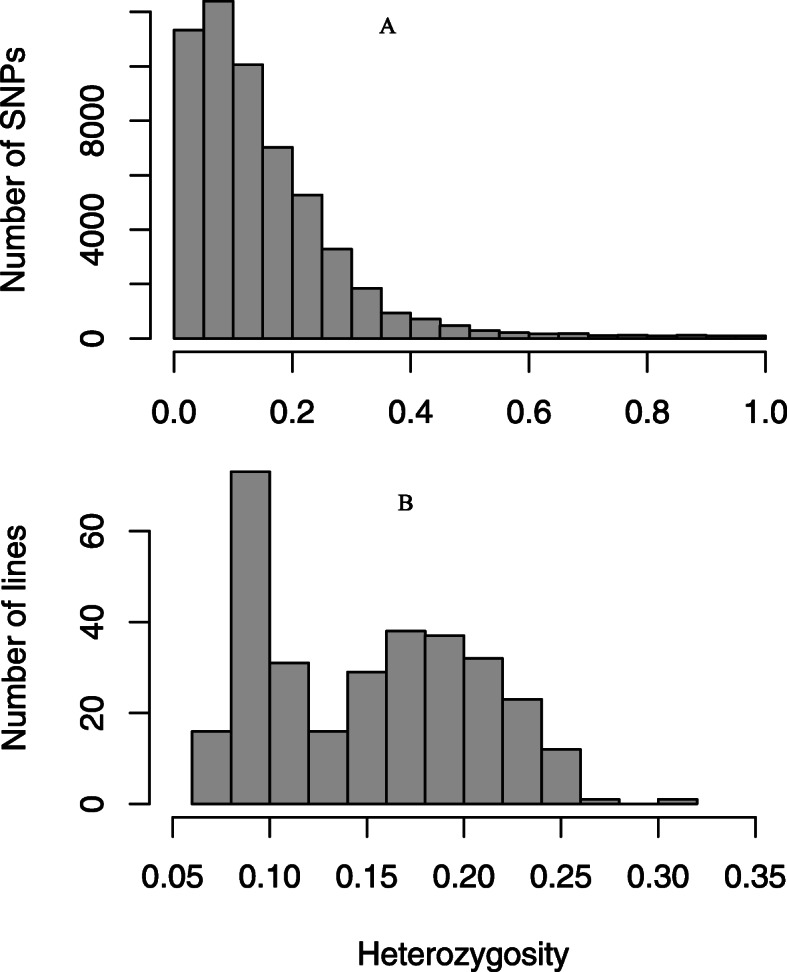
Single Nucleotide polymorphism (SNP) (**a**) and taxa heterozygosity (**b**) for 54,770 genome-wide SNPs markers detected among 309 pearl millet inbred lines

Among the five subgroups, subgroup 1 contained accessions such as ICTP 8203, GB8735, MC94, ICMV96490 and ICMR312, mainly known for their high levels of Fe and Zn content. Subgroup 2 was composed of landraces/improved cultivars mainly from WCA countries. Subgroup 3 was formed with inbred lines derived from landraces/improved cultivars collected from various sources, and showed more dispersion than the other subgroups. Results showed that the subgroup 4 contained inbred lines derived from the source populations IP-3110, IP-6745, IP-21142, PBPMPOP-1 and PBPMPOP-2, whereas group 5 comprised about 50% of inbred lines extracted from PBPMPOP-3.

### Allelic diversity and purity

In accordance with the neutral allele distribution expected for inbred populations, the nucleotide diversity (π) and Tajima’s D statistics among the five subpopulations were presented in Table 2. Degrees of SNP heterozygosity varied from 0 to 100% with an average of 15% (Fig. 6a). Most of the SNPs had a heterozygosity range of 0–25%. Similarly, genetic purity of the lines in the population varied from 70 to 93%, with a mean of 85% (Fig. 6b). Out of 309 inbred lines, 105 (34%) showed less than 10% heterozygosity, 145 (47%) had heterozygosity ranging from 11 to 20%, while 59 (about 20%) had heterozygosity from 21 to 30%.

**Table 2:** The nucleotide diversity (π) and Tajima’s D statistics among 309 pearl millet inbred lines grouped into five subpopulations

Population/Group	Nucleotide diversity (π)			Tajima’s D		
	Mean	Min	Max	Mean	Min	Max
Whole Population	9.92E-06	1.32E-07	5.89E-05	2.383	−0.367	5.102
Subgroup 1	8.78E-06	1.16E-07	5.13E-05	0.804	−1.833	3.304
Subgroup 2	9.77E-06	1.26E-07	5.65E-05	1.857	−1.237	4.227
Subgroup 3	9.25E-06	1.50E-07	5.58E-05	0.845	−1.452	3.274
Subgroup 4	9.24E-06	7.14E-08	5.61E-05	0.713	−1.687	3.149
Subgroup 5	7.32E-06	1.00E-07	4.90E-05	0.269	−1.897	2.477

### Genetic differentiation

Genetic differentiation of subgroups was calculated using F_st_-based analysis of the SNP data (Table 3). The *Fst* coefficients showed that subgroup 2 could be considered as a subset of the whole panel used in this study with a very low *Fst* value as compared to the whole population (0.002). The *Fst* coefficients among the groups varied from 0.044 to 0.110, indicating moderate differentiation. The highest level of differentiation was observed between subgroups 4 and 5. In contrast, the lowest *Fst* coefficient was observed between subgroups 2 and 3.

**Table 3:** F_ST_ among the five sub-populations determined by population structure analysis and the whole panel

	Population	Subgroup 1	Subgroup 2	Subgroup 3	Subgroup 4
Subgroup 1	0.027				
Subgroup 2	0.002	0.040			
Subgroup 3	0.025	0.062	0.044		
Subgroup 4	0.044	0.090	0.055	0.071	
Subgroup 5	0.052	0.086	0.066	0.076	0.110

The mean nucleotide diversity observed in subgroup 2 (9.77E-06) was similar to the whole panel (9.92E-06) (Table 3). The nucleotide diversity among accessions within the subgroups ranged from 7.32E-06 to 9.77E-06. Accessions from Group 5 showed lowest diversity in the population. The mean Tajima’s D values were positive for all the subgroups, which is an indication for balancing selection.

### Linkage disequilibrium

**Fig. 7:**
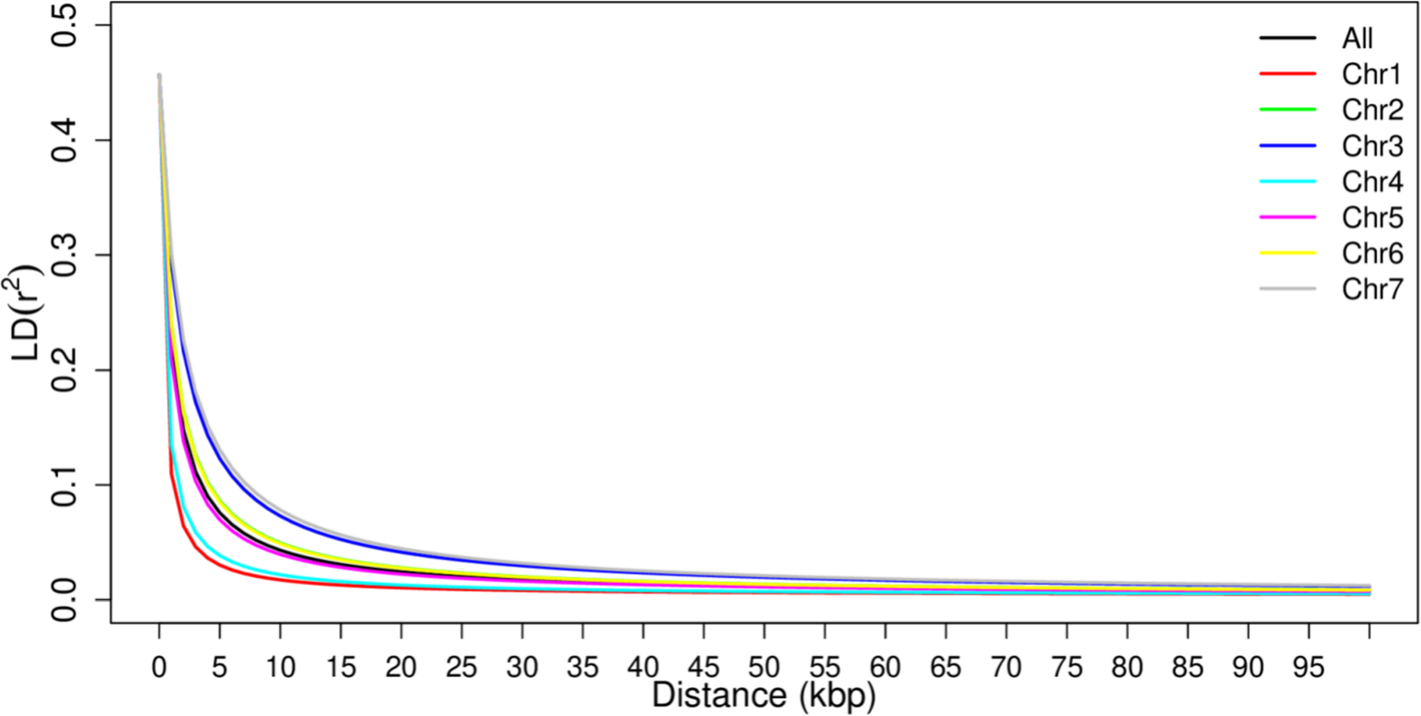
Chromosome-based LD (r2) decay of seven chromosomes in 309 pearl millet inbred lines using 54,770 high quality SNP markers developed using genotyping-by-sequencing (GBS)

LD among the SNPs was investigated between pairs of SNP markers from the seven chromosomes and then between pairs of SNP markers from the same chromosome. The average pairwise LD (r^2^) across the genome declined rapidly with increasing physical distance and most of the r^2^ values were below 0.05 (Fig. 7). On average, LD declined from its initial value of 0.46 to 0.1 within approximately 3.5 kb. LD decays rapidly in chromosomes 1 and 4 (~ 1.5 kb) as compared to 3 and 7 (~ 7.2 kb), suggesting that a larger number of markers are required from chromosomes 1 and 4 than from chromosomes 3 and 7 for genome-wide association studies in pearl millet.

## Discussion

SNPs have become the markers of choice in genetics and evolutionary biology studies, as well as in applications for marker-assisted selection in plant breeding. High-density of markers on a large numbers of individuals is vital for precise quantitative trait locus (QTL) mapping and association analysis [4]. GBS is a NGS based genotyping platform [14] that has the advantage of reduced genome representation to enable high-throughput genome-wide SNP genotyping with an affordable cost.

In this study, 309 pearl millet inbred lines were genotyped using GBS. Using the pearl millet genome [37] as a reference and filtering the dataset resulted in the development of 54,770 high quality genome-wide SNPs. The level of heterozygosity of the SNPs ranged from of 0 to 20% with an average of 15%. Since we pooled 4–6 plants for genomic DNA extraction, this level of heterozygosity may be attributed to the heterogeneity of plants within an accession. It has also been reported that high outcrossing rates, a sequencing error or mapping error may lead to high heterozygosity in pearl millet [19]. The level of homozygosity of the genotypes ranged from 70 to 93%, with an average of 85%, which is expected for inbred lines.

Genome-wide marker density analysis across the chromosome arms identified an average of 35 SNPs per Mb (1 SNP per 29 Kb) of genome size. This SNP density is slightly lower than the previously reported 48 SNPs per Mb of genome [32]. The report revealed that the distribution of the SNPs was dense in the telomeric regions than the pericentromeric regions of the pearl millet chromosomes probably because of low recombination rates, low gene density and/or low restriction sites for the enzymes around the centromere. In pearl millet, the location of each centromere has not been determined. Therefore, the low SNP density in one arm of chromosome 5 is possibly associated with the location of centromere. This phenomenon can also be attributed to decreased restriction enzyme sites in this genome region, or fewer polymorphisms related to interactions of different causes of genetic variation such as mutation, selection, recombination, and genetic-drift which shape nucleotide polymorphisms across the genome [3, 10, 11, 16]. Low gene density, which is associated with low nucleotide diversity [15], and decreased natural and artificial selection for alleles located in this part of the genome are both additional plausible reasons for lower marker numbers.

Germplasm resources and the genetic diversity of a crop species have paramount importance in the genetic improvement of a crop for desirable traits and conservation of genetic resources. Nucleotide polymorphism is a measure of genetic diversity and a key to understanding the effect of past selective forces on the gene pool. Average nucleotide diversity in the whole panel was 0.28 in this study, which was higher than reported for a global collection [19], but lower than the mean gene diversity (0.54) estimated using simple sequence repeat (SSR) markers in a pearl millet inbred germplasm association panel (PMiGAP) [31]. As adaptive evolution is implicated in reducing functional diversity [18], genetic distance among inbred lines derived from landraces grown in similar environments is expected to be low. Nevertheless, ecology and evolution work together to determine the population stability and maintain diversity within and among populations [21].

Population structure is a very important part of evolutionary genetics and depicts the diversity of a metapopulation that might have evolved independently. Knowledge about the genetic diversity and the population structure of a crop has important implications for a genome-wide association study. In the present study, population structure analysis using 30,893 SNPs detected five subgroups in a panel of 309 inbred lines and the grouping basically matches pedigree relationships or the parental source of the inbred lines. This number of subpopulations was validated by graphing kinship against the cross-validation error. Some genetic diversity studies reported six subpopulations in different panels of pearl millet [31, 32]. A population genomics study conducted on a collection of landraces from Senegal in comparison with a global collections, observed more diversity in the former [19], settling the West African origin of pearl millet [8]. Multiple factors such as natural selection, migration, and genetic drift might be the mechanisms that caused changes in allele frequencies over time and acted as forces for genetic diversification and population structure formation [9]. Grouping of pearl millet inbred lines from the same geographic region into different subpopulations implies that selection for different traits is maintaining genetic diversity.

A wide range of genotypic variations are prevalent in pearl millet for various agronomic traits and stress tolerance as a result of its cultivation in diverse agro-climatic conditions and soil types [34]. However, a limited amount of germplasm has been exploited in breeding to improve its agronomic traits, stress tolerance, and productivity in pearl millet [28]. Only a limited number of studies have assessed the evolutionary dynamics and genetic diversity patterns in pearl millet. Genetic characterization of early- and late-flowering landraces from Senegal also indicated a large diversity in Senegalese pearl millet germplasm that may be useful in defining heterotic groups and formation of a genomic association panels for trait mapping [13]. This study provides a survey of genetic variation in pearl millet inbred lines from different geographic regions in Africa and Asia representing various agroecological niches. However, as the inbred lines were developed from landraces, improved varieties, and crosses between different genotypes, correlation of the subgroups to geographic origin could not be made.

Genetic patterns in the natural plant populations shows their biological importance in the ecology and economic prominence in agriculture [24]. In pearl millet, genetic differentiation is important in the genetic improvement of the crop for productivity and adaptation in the agriculturally marginal environments. The genetic differentiation observed in the current study is the reflection of the extent of genetic variation among the landraces and improved varieties grown in different areas. The population structure analysis revealed five subgroups that mostly followed pedigree differences. As pedigree is the reflection of geographic origin of the parents involved and selection history of the inbred lines, growing environment may had significant role in the formation of distinct forms that are distantly related. However, the *F*_*st*_ values between the subgroups showed moderate differentiation. A previous study conducted in Niger revealed also a moderate level of differenciation on cultivated pearl millet accessions compared to the wild populations [25]. This variation at the DNA sequence level is important in the formation of preliminary heterotic groups through intra and inter-cluster crossing and subsequent studies to maximize hybrid performance.

## Conclusions

A better understanding of genetic diversity in pearl millet enhances the use of available germplasm for breeding and systematic conservation. Screening 309 pearl millet lines with 54,770 high quality genome-wide SNPs revealed that genetic diversity is preserved in pearl millet inbred lines from Africa and the Indian subcontinent. Population structure analysis detected five subpopulations that are mostly a reflection pedigree relationship. Population diversity analysis using *F*_*st*_ revealed moderate differentiation among the five subpopulations. The nucleotide diversity and Tajima’s D statistics showed low diversity in subgroup 5, but high diversity in the other four subgroups. The genomic resources developed in this study can significantly contribute to the application of genomic-assisted breeding in pearl millet, especially in heterotic grouping and hybrid breeding.

## Methods

### Plant materials

A total of 309 inbred lines obtained from International Crop Research in Semi-Arid Tropics (ICRISAT-Niger) were used in this study (Additional file S1). The inbred lines were developed following 4 to 6 generations of selfing of landraces originating from WCA countries and Asia, and improved open pollinated varieties (OPV) with improved iron (Fe) and zinc (Zn) content.

### DNA extraction, library construction, and genotyping

The seeds of the inbred lines were planted in a 96-cell trays in a greenhouse at Kansas State University. Fresh leaf tissues pooled from four to six seedlings per line were collected 7 days after planting, and freeze-dried for 48 h to remove water rapidly. Genomic DNA was extracted following a modified 2% CTAB protocol as previously described [32]. DNA was quantified using Quant-iT™ PicoGreen® dsDNA assay kit (Thermo Scientific, Waltham, MA, USA) on a FLUOstar Omega fluorescence plate reader (BMG LABTECH, Cary, NC, USA) and normalized to 25 ng/ul with 10 mM TRIS.

For library construction, 200 ng of DNA was double digested with *PstI* (5′-CTGCA/G-3′) and *MspI* (5′-C/CGG-3′) restriction enzymes (New England Biolabs, Ipswitch, MA, USA). Then the GBS [14] libraries were constructed following Mascher et al. [26]. The DNA fragments from each sample were ligated to unique barcoded-adapters for identification and multiplexing of samples for DNA sequencing and analysis. GBS libraries were sequenced twice on an Ion Torrent Proton Sequencer (ThermoFisher Scientific, Waltham, MA, USA) at the USDA-ARS Central Small Grain Genotyping Laboratory at Kansas State University, Manhattan, Kansas.

### SNP calling and filtering

The sequencing runs generated a total of 750.2 million reads. The pearl millet reference genome (https://cegresources.icrisat.org/data_public/PearlMillet_Genome/v1.1) [37] was used to map GBS reads and identify SNPs using TASSEL 5.0 GBSv2 pipeline, (www.maizegenetics.net) [6]. The mapping of reads to the pearl millet reference genome was done using BWA version 0.7.17-r1188 [23]. An 80 bases poly-A tail was added to the 3′ end of all the sequencing reads to avoid discarding short reads. The alignment detected a total of 150,977 raw SNP data points with a minimum SNP locus coverage of 0.19 and a minimum minor allele frequency of 0.0097. Then, the SNPs were filtered to remove those with unknown locations, indels, a minor allele frequency (MAF) of < 5% and a maximum missing data of 20%. Missing data for the remaining 115,772 SNPs were imputed using Beagle V5.1 [7] using the default parameters. A total of 54,770 SNPs (47% of the initial SNPs data points) were used for further statistical analyses. Population structure and PCA analysis were conducted with 30,208 evenly distributed SNPs that were randomly selected from the filtered SNP dataset.

### Data analysis

Genotype and taxa summaries (markers distribution, MAF, SNP heterozygosity, genetic distance, genetic purity, pairwise comparison and Kinship matrix) were calculated using TASSEL v.5 software [6]. Population structure of the SNP genotype datasets was analyzed using ADMIXTURE software [1] for K = 1 to 10. An accession was assigned to a subpopulation when the proportion of coefficient of membership to a subpopulation was greater than 60%. To confirm the admixture results, PCA was performed using the snpgdsPCA function of the R package SNPRelate [41].

LD analysis was performed using TASSEL as a standardized disequilibrium coefficient (D’) [17] and squared allele-frequency correlations (r^2^) [40] among pairs of loci. LD decay was fitted using a nonlinear *ls* function in R. Genetic difference among the identified subgroups were determined using *Fst* across the SNPs as a measure of population differentiation due to genetic structure. DNA sequence variation within and between populations was estimated by analyzing the genetic diversity (pi) and Tadjima’s D [35] in a sliding window size of 1 Mbp.

## Supplementary information

**Additional file 1.** List of materials with their pedigree and origin.

**Additional file 2.** SNP markers (54,770) developed for 309 pearl millet inbred lines.

**Additional file 3.** Relative kinship among pairs of inbred lines. (CSV 1417 kb)

**Additional file 4.** Genetic distance among pairs of inbred lines. (CSV 1611 kb)

## Data Availability

All the raw sequencing reads for all the accessions have been submitted to the National Centre for Biotechnology Information (NCBI) sequence read archive and deposited under the accession ID or “BioProject ID”: PRJNA598172. The SNP markers developed in this study were included as a supplementary information with this paper (Additional file S2).

## Abbreviations

A: Adenine
C: Cytosine
Chr: Chromosome
CTAB: Cetyl Trimethyl Ammonium Bromide
DNA: Deoxyribonucleic Acid,
Fe: Iron
G: Guanine
GBS: Genotyping-by-sequencing
GD: Genetic distance
ICRISAT: International Crops Research Institute for the Semi-Arid Tropics
LD: Linkage disequilibrium
MAF: minor allele frequency
NCBI: National Center for Biotechnology Information
NGS: Next-generation sequencing
NJ: Neighbor joining
OPV: Open pollinated variety
PCA: Principal component analysis
QTL: Quantitative trait locus/loci
SNP: single ne
T: Thymine
TRIS: Tris (hydroxymethyl) aminomethane
USDA-ARS: United States Department of Agriculture-Agricultural Research Service
WCA: West and Central Africa
Zn: Zinc
